# Synthesis of Ethano-Bridged Diazapolycenes as Potential Precursors for Diazapolycenes and Their Properties

**DOI:** 10.3390/molecules21040407

**Published:** 2016-03-25

**Authors:** Moinul Karim, Yurngdong Jahng

**Affiliations:** Department of Pharmacy, College of Pharmacy, Yeungnam University, Gyeongsan 38541, Korea; mukulnmr@gmail.com

**Keywords:** azapentacene, diazapolycene, 6,13-dihydro-6,13-ethano-5,12-diazapentacene, 7,16-dihydro-7,16-ethano-6,15-diazaheptacene, 8,19-dihydro-8,19-ethano-7,18-diazanonacene

## Abstract

A series of ethanodiazapolycenes were prepared in 87%–89% yields by Friedländer reactions of three *o*-aminoarenecarbaldehydes with bicyclo[2.2.2]octane-2,5-dione and their spectral, thermal, and structural properties were studied. Subsequent attempts to convert them to diazapolycenes have proved unsuccessful.

## 1. Introduction

**Figure 1:**
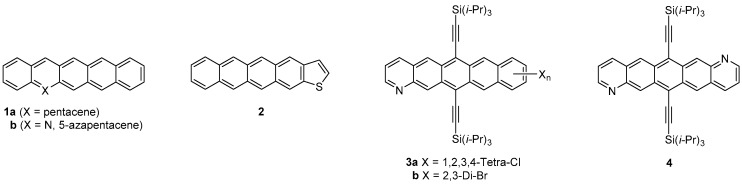
Pentacene and its isosteres.

The polycenes, represented by pentacene (**1a**) have long been of interest in the area of semiconductors with potential applications in organic thin-film transistors (OTHFTs) [1,2,3], and organic light-emitting diodes [4]. In order to overcome the major drawbacks of pentacene such as its insolubility at room temperature and rapid degradation [5], continuous efforts have been pursued to prepare soluble precursors that can be thermally converted to pentacene [6], the introduction of substituent(s) on the basic skeleton [7,8,9], and substitution of the benzene moiety by isosteric aromatics such as thiophene (compound **2**) [10], and pyridine (compounds **3** [11] and **4** ([12]) (Figure 1).

Theoretically, introduction of a nitrogen atom significantly decreases the frontier molecular orbital energy and hence improves the stability in air and to light [13]. This has led to the synthesis of large numbers of *N*-heteroacenes with pyrazine units in recent years [14,15]. The 1-azapentacenes with bis(triisopropylsilyl)ethynyl groups such as **3** in fact showed potential for high-performance organic semiconductors with superior structure-activity characteristics that the corresponding pentacene derivative, 6,13-bis[(triisopropylsilyl)ethynyl]pentacene [16]. Recently, we reported a synthesis and the properties of 5-azapentacene (**1b**, Figure 1) [17], which might open a new vista for the studies on azapolycenes. As part of our ongoing studies on azaaromatics [17,18,19], we describe herein a preparation of ethano-bridged diazapolycenes as dimeric diazapolycenes and our attempts at the preparation of the corresponding diazapolycenes.

## 2. Results and Discussions

### 2.1. Synthesis 

**Scheme 1:**
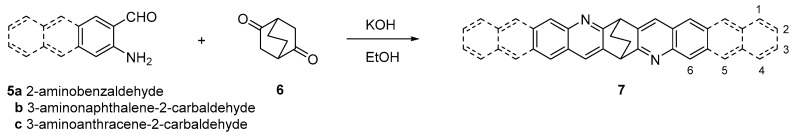
Synthesis of ethano-bridged diazapolycenes.

Synthesis of the compounds was straightforward as shown in Scheme 1. The Friedländer reactions of *o*-aminoarenecarbaldehydes **5** with diketone **6** afforded the corresponding ethano-bridged diazapolycenes **7** in 87%–89% yields. The prerequisite starting 2-aminobenzaldehyde (**1a**) [20], 3-aminonaphthalene-2-carbaldehyde (**5b**) [21], 3-aminoanthracene-2-carbaldehyde (**5c**) [17], and bicyclo[2.2.2]octane-2,5-dione (**6**) [22] were prepared employing the corresponding previously reported methods.

### 2.2. Attempts to Convert ***7*** to the Corresponding Diazapolycenes ***8***

**Scheme 2:**
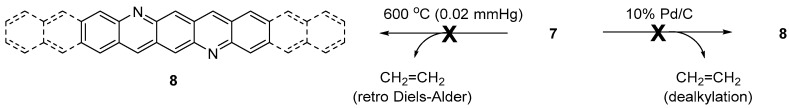
Synthesis of diazapolycenes.

Catalytic dehydrogenation and/or dealkylation [23,24,25,26] and vacuum pyrolysis [27,28,29,30,31] of cyclohexenes have been commonly employed to build up benzene rings, in which the former proceeds via elimination of alkyl substituent(s) and the latter via retro-Diels-Alder reaction. However, all attempts, including catalytic dehydrogenation (10% Pd/C in nitrobenzene at 200 °C for 8 h) and vacuum pyrolysis (0.02 mmHg, 600 °C, 45–60 min) of the compounds **7** leading to the corresponding diazapolycenes **8** have been as yet unsuccessful (Scheme 2). It should be noted that vacuum pyrolysis of **7a**,**b** resulted in sublimation of the compounds which remain unchanged, while **7c** decomposed.

### 2.3. Spectroscopic Properties

^1^H-NMR spectral data of selected protons are summarized in Table 1. All the proton resonances were assigned by comparison with previously reported data for related compounds such as quinoline [32], benzo[*g*]quinoline [33], and 5-azapentacene [17] and by double quantum H-H COSY experiments. The resonances of H4 and the proton at the *peri*-position in the most of the polypyridines and polyquinolines, are characteristic and have been used as a diagnostic probe for understanding the structural information. The resonances of H4 were shifted downfield by 0.73 ppm for **7b** compared to **7a**, and 0.40 ppm for **7c** compared to **7b**. These values are comparable to those of related compounds such as quinoline, benzo[*g*]quinoline, and 5-azapentacene, reflecting increased delocalization of π-orbitals in the aromatic systems as the number of aromatic rings increases. Similar trends were also observed for the proton at the *peri*-position. It should be noted that the ethane bridge protons of **7a** resonated at δ 2.03 as a singlet, while those of **7b** and **7c** were multiplets in the range of δ 2.25–2.10 and δ 2.27–2.23, respectively, reflecting the fact these bridges are rigid at room temperature on the NMR time scale.

**Figure 2:**
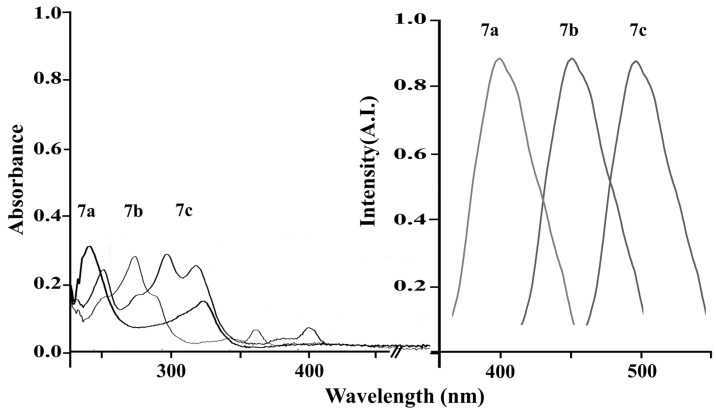
UV absorption and photoluminescence (PL) spectra of **7a**, **7b**, and **7c** in EtOH (1 × 10^−6^ M) at 298 K.

UV absorption spectra of **7** were obtained in EtOH (1.0 × 10^−6^ M) (Figure 2) and the absorption maxima and extinction coefficients are summarized in Table 2. The absorption bands in the 245–303 and 331–400 nm regions correspond quite closely to the *π*-π* absorptions. The absorption maxima for the more linear compounds (**7a** *vs.* **7b**, **7b** *vs.* **7c**) appear shifted towards longer wavelengths (see Figure 2). These data are consistent with an electronic transition state in which the energy of the receptor π* orbital is lowered by the increasing delocalization which would be found for the more conjugated and linear systems. The electronic spectrum of the most linear compound **7c** showed *π*-π* absorption bands at 303 and 325 nm which are red-shifted compared to the parent compound **7a** by 58 and 80 nm, with little effect on the intensity of the absorption compared to **7a**. It should be noted that benzoannulation led an additional absorption band in the region of 350–408 nm, of which **7c** showed the most intense and bathochromic shifted absorption due to the additional benzene ring.

The photoluminescence (PL) of the compounds was studied in EtOH (1 × 10^−5^ mol/L) at room temperature (Figure 2) and are summarized in Table 2. Excitation of the absorbances at 245, 280, and 303 nm for **7a**, **7b**, and **7c**, respectively, showed emissions at 400, 465, and 500 nm. The observed emission wavelength is somewhat dependent on the nature of the conjugated system: The most extended compound **7c** showed a green light emission at 500 nm while **7a** showed a purple light emission at 400 nm. The quantum yields were determined by a previously reported method [34,35] employing quinine sulfate as a standard to give values of 0.38, 0.54, and 0.58 for **7a**, **7b**, and **7c**, respectively.

### 2.4. Structural and Thermal Properties

**Figure 3:**
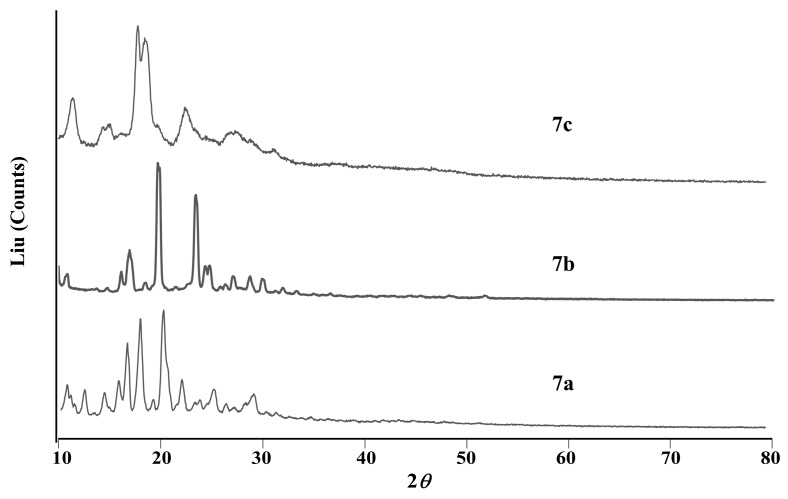
X-ray diffractograms of compounds **7a**, **7b**, and **7c** in powder state.

The crystallinity of the compounds prepared was analyzed by X-ray diffraction (XRD) and the corresponding X-ray diffractograms are shown in Figure 3. The diffractograms of compounds **7a** and **7b** showed distinctive peaks, indicating their crystalline nature. Compounds **7a**, of which the X-ray crystal structure has been reported [36], and **7b** have more crystalline character comparing to **7c**. The crystallite sizes of **7a** and **7b** were calculated by employing Scherrer’s equation, *D*_p_ = 0.93λ/*L*cosθ [37], where *D*_p_ is the averaged particle size of the crystallites, λ is the incident wavelength (1.54056 Å), θ is the Bragg angle and *L* is the diffracted full width at half maximum (in radians) caused by crystallization to give 6.89 and 10.54 nm for **7a** and **7b**, respectively.

The thermal behaviors of the compounds were analyzed by differential scanning calorimetry (DSC). All the compounds showed a single sharp endothermic peak at the melting transition temperature (*T*_m_) of 287.3, 397.35, and 409.78 °C, respectively. However only compound **7a** showed sharp exothermic peaks as a crystallization temperature (*T*c) at 202.74 °C (data not shown). It should be noted that none of the compounds showed a glass transition temperature (*T*_g_).

## 3. Experimental Section

### 3.1. General Information

Melting points were determined using a Fischer-Jones melting points apparatus (Fischer Scientific, Grand Island, NY, USA) and are not corrected. UV spectra were recorded on a V550 spectrophotometer (JASCO, Oklahoma City, OK, USA). NMR spectra were obtained using a Bruker-250 spectrometer (Fällanden, Switzerland) or VNS600 FT-NMR (Varian, Australia) operating at 250 MHz or 600 MHz for 1H-NMR and 62.5 MHz or 150 MHz for 13C-NMR and are reported as parts per million (ppm) from the internal standard TMS. Chemicals and solvents were commercial reagent grade and used without further purification. Electrospray ionization (ESI) mass spectrometry (MS) experiments were performed on a LCQ advantage-trap mass spectrometer (Thermo Finnigan, San Jose, CA, USA). Elemental analyses were taken on a Hewlett-Packard Model 185B elemental analyzer (Hewlett Packard, Littleton, MA, USA). XRD analysis was performed by X-ray diffractometry (MPD for bulk, PANalytical, Wesybrough, MA, USA) with nickel-filtered CuKα radiation (30 kV, 30 mA) at 2θ angles from 10° to 90°, a scan speed of 10°/min and a time constant of 1 s. Thermal behaviors of the compounds were analyzed using differential scanning calorimeter (DSC Q200, TA Instrument, Wilminton, NJ, USA) with 1~2 mg of sample sealed in alumina in the range of 40–385 °C increasing temperature in a rate of 10 °C/min. An empty pan was used as a reference, and the DSC baseline, temperature, and enthalpy were calibrated. High and low resolution FAB-MS data were obtained on a JMS-700 instrument (JEOL, Peabody, MA, USA).

### 3.2. Synthesis and Characterization of the Products

#### *6,13-Dihydro-6,13-ethano-5,12-diazapentacene* (**7a**)

To a mixture of 2-aminobenzaldehyde (**5a**, 0.59 g, 4.9 mmol) and bicyclo[2.2.2]octane-2,5-dione (**6**, 0.30 g, 2.18 mmol) in EtOH (15 mL) was added 2 M KOH in EtOH (1 mL). The resulting mixture was refluxed for 8 h to form a precipitate which was collected and washed with EtOH to give pale yellow needles (0.60 g, 88%): mp 297–298 °C (lit. [36] mp 284–285 °C). Spectral data were identical to those reported previously [36]. ^1^H-NMR (CDCl_3_, 250 MHz) δ 8.33 (s, 2H, H7 and H14), 8.01 (d, *J* = 8.3 Hz, 2H, H4 and H11), 7.95 (d, *J* = 7.9 Hz, 2H, H1 and H8), 7.73 (td, *J* = 8.3, 1.0 Hz, H3 and H10), 7.60 (td, *J* = 8.0, 1.0 Hz, H2 and H9), 4.80 (s, 2H, H6 and H13), 2.03 (s, 4H). ^13^C-NMR (62.5 MHz, CDCl_3_) δ 25.5, 45.8, 126.3, 127.3, 128.0, 128.6, 129.5, 130.3, 134.2, 146.3, 163.4.

#### *7,16-Dihydro-7,16-ethanobenzo[b]benzo[6,7]quinolino[3,2-i]acridine (7,16-dihydro-7,16-ethano-6,15-diaza-heptacene,* **7b**)

To a mixture of 3-aminonaphthalene-2-carbaldehyde (**5b**, 280 mg, 1.63 mmol) and bicyclo[2.2.2]octane-2,5-dione (**6**, 90 mg, 0.65 mmol) in EtOH (10 mL) was added 2 M KOH in EtOH (1 mL). The resulting reaction mixture was refluxed for 8 h. Evaporation of the solvent provided pale yellow crystalline solids (236 mg, 89%), which was purified by flash column chromatography on silica eluting with EtOAc to give the desired product as pale yellow needles: mp 397.35 °C (DSC). ^1^H-NMR (CDCl_3_, 250 MHz) δ 8.60 (s, 2H, H5 and H14), 8.33 (s, 2H, H8 and H17), 8.19 (s, 2H, H9 and H18), 8.03 (overlapped dd, 4H, *J* = 7.8, 2.8 Hz, H1, H4, H10, H13), 7.50–7.46 (m, 4H, H2, H3, H11, H12), 4.77 (s, 2H, H7 and H16), 2.25–2.10 (m, 4H). ^13^C-NMR (CDCl_3_, 62.5 MHz) δ 163.9, 143.6, 133.8, 133.1, 131.8, 130.6, 128.6, 128.2, 126.7, 126.6, 126.4, 126.3, 126.1, 46.7, 25.5. MS (ESI) calcd for C_30_H_20_N_2_ [M + H]^+^ 409, found 409. HR-FAB-MS (*m*/*z*): [M^+^] calcd for C_30_H_20_N_2_, 408.16265; found, 408.1629. Anal. calcd for C_30_H_20_N_2_ C, 88.21; H, 4.94; N, 6.86. Found C, 88.39; H, 4.93.

#### *8,19-Dihydro-8,19-ethanonaphtho[2,3-b]naphtho[2′,3′:6,7]quinolino[3,2-i]acridine (8,19-dihydro-8,19-ethano-7,18-diazanonacene,* **7c**)

A mixture of 3-aminoanthracene-2-carbaldehyde (**5c**, 360 mg, 1.63 mmol, 2.5 equiv), bicyclo[2.2.2]octane-2,5-dione (**6**, 90 mg, 0.65 mmol), and 2 M KOH in EtOH (1 mL) in EtOH (10 mL) was refluxed for 8 h. Work-up as described above for **7a** afforded a pale crystalline solid (291 mg, 88%) as a desired product which was flash-chromatographed on silica eluting with EtOAc. The later fractions afforded yellow solid: mp 409.78 °C (DSC) (dec). ^1^H-NMR (CDCl_3_, 250 MHz) δ 8.85 (s, 2H, H6 and H17), 8.73 (s, 2H, H9 and H20), 8.68 (s, 2H, H10 and H21), 8.59 (s, 2H, H5 and H16), 8.21 (s, 2H, H11 and H22), 8.03 (dd, 2H, *J* = 7.8, 1.5 Hz, H4, H14), 8.00 (dd, 2H, *J* = 7.8, 1.5 Hz, H1, H12), 7.45–7.41 (m, 4H, H2, H3, H13, H14), 4.76 (s, 2H, H8 and H18), 2.27–2.23 (m, 4H). A ^13^C-NMR spectrum could not be recorded due to the low solubility in common NMR solvents. MS (ESI) calcd for C_38_H_25_N_2_ [M + H]^+^ 509, found 509. HR-FAB-MS (*m*/*z*): [M^+^] calcd for C_38_H_24_N_2_, 508.19395; found, 508.1641. Anal. calcd for C_38_H_24_N_2_-1.5H_2_O C, 85.21; H, 5.08; N, 5.23. Found C, 85.26; H, 5.07; N, 5.21.

### 3.3. Attempted Synthesis of Diazapolycenes ***8***

*Method A:* A mixture of **7** (1.0 mmol) and 10% Pd/C (100 mg) in nitrobenzene (10 mL) was heated 200 °C for 8 h. After cooling the reaction mixture to room temperature, reaction mixture was filtered through Celite^®^. Work up as usual afforded a pale yellow solid, which turned out the starting material based on TLC as well as ^1^H-NMR.

*Method B:* The vacuum pyrolysis (0.02 mmHg, 600 °C, 45–60 min) of the compound **7** (1.0 mmol) did not lead to the corresponding diazapolycenes **8** but instead sublimation of the product occurred for **7a**,**b**, while **7c** was decomposed at 409 °C without sublimation.

### 3.4. Measurement of Quantum Yield (Φ)

The quantum yield of the compounds prepared was measured by using a quinine sulfate solution (in 0.1 M H_2_SO_4_, literature quantum yield 0.577 at 360 nm [34,35]) as the standard and calculated with the equation of Φ = Φ_R_ × (I/I_R_) × (OD_R_/OD) × (*n*^2^/*n*_R_^2^), where Φ is the quantum yield, I is the measured integrated emission intensity, *n* is the refractive index, OD is the optical density and the subscript R refers to the quinine sulfate.

## 4. Conclusions

The Friedländer reactions of three *o*-aminoarenecarbaldehydes, namely 2-aminobenzaldehyde, 3-aminonaphthalene-2-carbaldehyde and 3-aminoanthracene-2-carbaldehyde, with bicyclo[2.2.2]-octane-2,5-dione yielded a series of ethanodiazapolycenes as dimeric azapolycenes in 87%–89% yields and their spectral, thermal, and structural properties were studied. All attempts to further convert the products into diazapolycenes by *retro*-Diels-Alder reaction or vacuum pyrolysis were not successful.

## Figures and Tables

**Table 1:** ^1^H-NMR chemical shifts and coupling constants of selected protons.

Compounds	H4	H at *Peri*-Position	-CH_2_CH_2_-
Quinoline ^(a)^	8.00 (^3^*J* = 8.3, ^4^*J* = 1.1 Hz)	8.05 (^3^*J* = 8.2, ^4^*J* = 1.6 Hz)	
Benzo[*g*]quinoline	8.58 (^3^*J* = 8.5, ^4^*J* = 1 Hz) ^(b)^ 8.31 (^3^*J* = 8.5 Hz) ^(c)^	8.76 (s) ^(b)^ 8.70 (s) ^(c)^	
Naphtho[2,3-*g*]quinoline ^(d)^	8.50 (^3^*J* = 7.5, ^4^*J* = 1.5 Hz)	8.87 (s)	
5-Azapentacene ^(e)^	9.12 (s)	9.26 (s)	
**7a**	7.60 (s)	8.01 (^3^*J* = 8.3, ^4^*J* = 1.2 Hz)	2.03 (s)
**7b**	8.33 (s)	8.59 (s)	2.25–2.10 (m)
**7c**	8.73 (s)	8.85 (s)	2.27–2.23 (m)

^(a)^ Ref. [32]; ^(b)^ Taken from 250 MHz ^1^H-NMR in CDCl_3_; ^(c)^ Ref [33]; ^(d)^ Taken from the ChemDraw^®^ computer program from CambridgeSoft; ^(e)^ Ref [17].

**Table 2:** UV absorption and FL data of compounds **7a**, **7b**, and **7c**.

Compd.	λ [nm (logε)]	λ_excit_	λ_emit_	Quantum Yield ^(a)^
**7a**	245 (5.49) 331 (5.18)	245	400	0.38
**7b**	256 (5.20, sh) 280 (5.46) 297 (5.20, sh) 350 (4.61) 370 (4.81)	280	465	0.54
**7c**	257 (5.39) 303 (5.46) 325 (5.41) 408 (4.85)	303	500	0.58

^(a)^ Values were calculated from the equation given in Ref. [34,35] using quinine sulfate as a reference.
